# Assessment of the Mechanisms of Action of Eribulin in Patients with Advanced Liposarcoma Through the Evaluation of Radiological, Functional, and Tissue Responses: A Prospective Monocentric Study (Malibu Study)

**DOI:** 10.3390/cancers17060976

**Published:** 2025-03-13

**Authors:** Maria Susanna Grimaudo, Federico D’Orazio, Salvatore Lorenzo Renne, Maurizio D’Incalci, Robert G. Maki, Piergiuseppe Colombo, Luca Balzarini, Alice Laffi, Armando Santoro, Alexia Francesca Bertuzzi

**Affiliations:** 1 Medical Oncology and Hematology Department, IRCCS Humanitas Research Hospital, Via Alessandro Manzoni 56, 20089 Rozzano, MI, Italy; alice.laffi@humanitas.it (A.L.); armando.santoro@humanitas.it (A.S.); alexia.bertuzzi@humanitas.it (A.F.B.); 2 Radiology Department, IRCCS Humanitas Research Hospital, Via Alessandro Manzoni 56, 20089 Rozzano, MI, Italy; federico.dorazio@humanitas.it (F.D.); luca.balzarini@humanitas.it (L.B.); 3 Pathology Department, IRCCS Humanitas Research Hospital, Via Alessandro Manzoni 56, 20089 Rozzano, MI, Italy; salvatore.renne@hunimed.eu (S.L.R.); piergiuseppe.colombo@hunimed.eu (P.C.); 4 Department of Biomedical Sciences, Humanitas University, Via Rita Levi Montalcini 4, 20090 Pieve Emanuele, MI, Italy; maurizio.dincalci@hunimed.eu; 5 Laboratory of Cancer Pharmacology, IRCCS Humanitas Research Hospital, Via Alessandro Manzoni 56, 20089 Rozzano, MI, Italy; 6 Memorial Sloan-Kettering Cancer Center, 1275 York Ave, New York, NY 10065, USA; makir@mskcc.org; 7 School of Medical Sciences, Weill-Cornell Medical College, 1300 York Ave, New York, NY 10065, USA

**Keywords:** eribulin, liposarcoma, DCE-MRI, vascularization, cellular differentiation

## Abstract

**Background:** Liposarcoma (LPS) is one of the most frequent histotypes of soft tissue sarcoma (STS). Eribulin is a cytotoxic agent that has improved overall survival in patients with advanced LPS. Additionally, preclinical and clinical evidence suggests its influence on vascularization and cellular differentiation. Based on these data, we developed this study to investigate non-mitotic effects of eribulin in patients with advanced LPS. **Methods:** In this prospective monocentric observational study, we included patients with advanced LPS eligible to receive eribulin. An assessment with dynamic contrast-enhanced magnetic resonance imaging (DCE-MRI) and a biopsy were planned before treatment and after four cycles of eribulin. DCE-MRI scans were elaborated to obtain perfusion and permeability maps. **Results:** From September 2019 to January 2024, 11 patients were enrolled. Among them, 8/11 (73%) had successful pre- and post-treatment assessment. At the time of the analysis, 8/11 (73%) patients had disease progression and 4 (36%) had died, median progression-free survival (mPFS) was 3.3 months, and median overall survival (mOS) was 8.7 months. Among the evaluable patients, DCE-MRI perfusion decreased after eribulin treatment in patients with disease control (partial response or stable disease), while perfusion values increased in patients with progressive disease (PD). No significant change in permeability was found. Post-treatment histological changes were seen nearly in all patients, with decreased cellularity the most common change (50%), followed by vascularization modifications (20%). **Conclusions:** Eribulin appears to exhibit non-mitotic activity involving both vascularization and cell differentiation in LPS patients. Further studies are needed to better define these effects.

## Simple Summary

Eribulin is an antimitotic agent approved for the treatment of patients with advanced liposarcoma. Preclinical studies suggest that it also has a complex antitumor activity, modifying vascularization and tumor cells differentiation in various cancer types. In our prospective study we enrolled patients with pretreated advanced well differentiated/dedifferentiated and myxoid liposarcoma eligible to receive eribulin and we performed a radiological and histological assessment before and after eribulin administration. According to the published data, we found that eribulin shows non-mitotic effects, conferring tumor control in a subgroup of patients with pretreated advanced liposarcoma. Dynamic contrast-enhanced magnetic resonance imaging (DCE-MRI) is a feasible technique to identify modifications in tumor vascularization.

## 1. Introduction

Sarcomas comprise a heterogeneous group of rare tumors [1], including more than 80 subtypes according to the 2020 WHO classification [2]. Within these different entities, liposarcoma (LPS) is one of the most common types, accounting for 15–20% of STSs [2,3,4].

LPSs are malignant adipocytic tumors classified into different variants based on specific biological, molecular and clinical features [4,5]. Well-differentiated liposarcomas (WDLPS) are low-grade malignant adipocytic tumors with overall good prognosis, while dedifferentiated liposarcoma (DDLPS) and pleomorphic liposarcoma (PLPS) represent less differentiated malignant tumors with poorer prognosis [2,5,6]. Myxoid liposarcoma (MLPS) encompasses about 30% of LPS, and the prognosis is related to the degree of histological hypercellularity [2].

Most studies on systemic treatments for advanced (unresectable or metastatic) LPS have been conducted on STSs as a whole, for which anthracyclines are seen as the most active chemotherapeutic agents [7,8,9]. Specifically, for LPS, MLPS exhibits better response rates and mOS than other LPS subtypes [10].

Beyond anthracycline-based treatment, subsequent options include ifosfamide, gemcitabine (+/− docetaxel), and dacarbazine, with limited benefit [4].

Among newer chemotherapeutic agents, trabectedin and eribulin have been approved for liposarcoma patients failing other therapies [11,12]. Eribulin mesylate (eribulin) is a synthetic molecule derived from the marine sponge *Halichondria okadai*, which exerts antimitotic activity by the binding to the positive ends of microtubules, causing mitotic arrest and apoptosis [13,14,15]. In addition, eribulin has shown different mechanisms of action including modifications of vascularization and tumor cell differentiation [14,15]. Clinical development in advanced breast cancer led to the pivotal EMBRACE trial, in which eribulin conferred an advantage in mOS despite not improving mPFS [16]. The phase 3 pivotal trial of eribulin on pretreated LPS and leiomyosarcoma patients documented improved mOS over dacarbazine (13.5 vs. 11.5 months), with identical mPFS (2.6 vs. 2.6 months) [12]. In the prespecified subgroup analysis, the advantage in OS was maintained only in patients with LPS (15.6 vs. 8.4 months, *p* < 0.001), not in the leiomyosarcoma cohort. This study led to the approval of eribulin in patients with advanced LPS.

We analyzed clinical, radiological, and pathological responses to eribulin in patients with LPS, introducing dynamic contrast-enhanced magnetic resonance imaging (DCE-MRI) as additional radiological assessment in the MALIBU study (Mechanisms of Action in patients with advanced LIposarcoma treated with EriBUlin), which was started in 2018.

## 2. Methods

### 2.1. Study Objectives

The primary objective was to investigate the mechanisms of action of eribulin in patients with advanced LPS through assessment of clinical, pathological, and radiological effects. The secondary objective was to identify possible factors that could have an impact on clinical outcomes (PFS and OS).

### 2.2. Study Design

This was a single center, prospective, observational study that included consecutive patients eligible to receive eribulin. We planned a radiological and histological assessment before the administration of eribulin and after four cycles (12 weeks) of eribulin therapy.

Radiological assessment included a CT scan and DCE-MRI covering the lesion chosen for biopsy. The histological assessment included a core biopsy of the selected lesion accounting for both technical aspects (guaranteeing patient safety) and radiological features suggestive of high-grade areas (value reduction in apparent diffusion coefficient (ADC) maps, high contrast enhancement, low T2 signal).

Eribulin was administered intravenously following the standard protocol of the pivotal trial [12] at 1.4 mg/m^2^ over 2–5 min at days 1 and 8 of 21-day cycles. Chemotherapy continued until clinical or radiological progressive disease with radiological evaluation every four cycles, while biopsy was only performed at first restaging.

The inclusion criteria were as follows:

- Histological diagnosis of liposarcoma
- Advanced stage (unresectable or metastatic) disease treated with at least two previous lines of chemotherapy (or ineligible for anthracycline and treated with at least a previous line of chemotherapy)
- Age ≥ 18 years
- Ability to give informed consent

The exclusion criteria were as follows:

- Prior eribulin treatment
- Patients unsuitable for biopsy due to medical conditions

In terms of statistical analyses, demographic, clinical, and biological characteristics of patients, as well as response, have been summarized as numbers and percentages or as median and range, as appropriate. All evaluations were considered exploratory in nature.

CT scan images were evaluated for response using Response Evaluation Criteria in Solid Tumors (RECIST) version 1.1 [17]. DCE-MRI images were obtained using 3T scanner (Siemens, Munich, Germany). To obtain dynamic vascularization data, fast scans with T1-weighted 3D fat saturated sequences were performed with isotropic voxels, repeated during contrast administration over 6 min scanning time. To quantify perfusion and permeability, DCE-MRI were analyzed through the software Olea Sphere (version 3.0.34). We collected quantitative data measuring the values for both parameters in four regions of interest (ROI) for each patient:

- biopsy site (precisely identified in patients who had CT scan-guided biopsies)
- “worst” tumor area with radiologic features suggestive of the most aggressive biology (reduction in ADC maps, high contrast enhancement, low T2 signal)
- “best” tumor area with the least aggressive radiologic features (lack of one or more aggressive features)
- a control area, identified as normal adipose tissue located in the same body region but separate from tumor tissue

Tissue specimens were obtained before and after treatment by core biopsy and formalin-fixed and paraffin-embedded. Hematoxylin/eosin-stained sections were submitted to morphologic evaluation of tumor tissue including histologic parameters such as cellular density, cellular atypia (pleomorphism), quantification of vascular structures, and quality of stroma.

This study was conducted in agreement with the Declaration of Helsinki and the laws and regulations of the country and according to the ICH Guideline for Good Clinical Practice. The protocol and its appendices were subject to review and approval by an Independent Ethics Committee(s) (“IEC”). All enrolled patients were informed of the aims of the study and provided written informed consent regarding the study and personal data protection.

## 3. Results

### 3.1. Patients’ Characteristics

From September 2019 to January 2024, 11 patients were enrolled (6 males and 5 females). Patient characteristics are summarized in Table 1. Median age at diagnosis was 59.2 years (range 27.8–80.0 years). A total of 9 out of 11 patients had DDLPS (82%; 1 with a prevalent WDLPS component), and 2/11 had MLPS (18%). All the assessable patients had G3 liposarcoma according to FNCLCC grading system [18], while histologic grade at diagnosis was not assessable for two patients because of an incomplete pathology report, though both had high grade primary tumors owing to the diagnosis of DDLPS. Upon diagnosis, all the patients presented with locally advanced disease and underwent surgery to remove the primary tumor. Regarding perioperative treatments, 27% of patients received perioperative radiotherapy and 45% perioperative chemotherapy.

Prior to eribulin, all patients had undergone at least two lines of chemotherapy for advanced disease, except for one patient that was ineligible for anthracyclines and was treated with trabectedin as the first-line treatment. Median time from diagnosis to the start of eribulin was 69.6 months (range 18–128 months). Most of the patients had undergone local treatments for recurrent/metastatic disease.

### 3.2. Clinical Evaluation

All the patients completed pre-treatment assessment and received at least one cycle of chemotherapy with eribulin. Within the included patients, 8/11 (73%) completed all assessments according to the study protocol. One patient received three cycles instead of four because of peripheral neuropathy that required treatment interruption of eribulin. A total of 3 out of 11 patients (27%) experienced clinical and radiological progression and did not participate in post-treatment assessment.

With a median follow-up of 9.2 months from the start of eribulin (range 1.3–38.5 months), 8/11 (73%) patients had disease progression and 4/11 (36%) died. In the overall population, median progression-free survival (mPFS) was 3.3 months, and median overall survival (mOS) was 8.7 months. Patients with disease control (stable disease (SD) or partial response (PR)) had longer mPFS (16.5 vs. 3.3 months) and overall survival (mOS 16.5 vs. 5.9 months) compared to patients with progressive disease (PD).

All the patients that had disease control or oligometastatic progression eligible for locoregional procedure continued treatment with eribulin, with a median duration of treatment of 3 months in the whole study population (range 1.5–36 months). One patient obtained PR after 8 months of treatment and underwent surgery to remove all visible tumor.

Treatment was generally well tolerated. Moderate-severe peripheral neuropathy was reported in two patients (one G2 peripheral neuropathy and one G3 peripheral motor neuropathy). Two patients developed G3 neutropenia. No other >G1 toxicities were observed.

### 3.3. Radiological Evaluation

#### 3.3.1. RECIST Assessment (CT Scan)

A total of 8/11 patients (73%) had progressive disease (PD), 2 (18%) had SD, and 1 had PR per RECIST 1.1. [17]

Patient M09 was declared as PD at first assessment because of the detection of a new abdominal lesion, while the known target lesions remained stable according to RECIST. This patient agreed to treatment beyond progression, and continued eribulin with ablative stereotactic body radiotherapy (SBRT) on the new lesion.

#### 3.3.2. DCE-MRI Assessment

Among the eight patients that underwent both pre- and post-treatment DCE-MRI, four patients (50%) had PD, three (38%) had SD (including patient M09), and one (13%) had PR. Descriptive DCE-MRI evaluation is detailed in Table 2.

**Figure 1:**
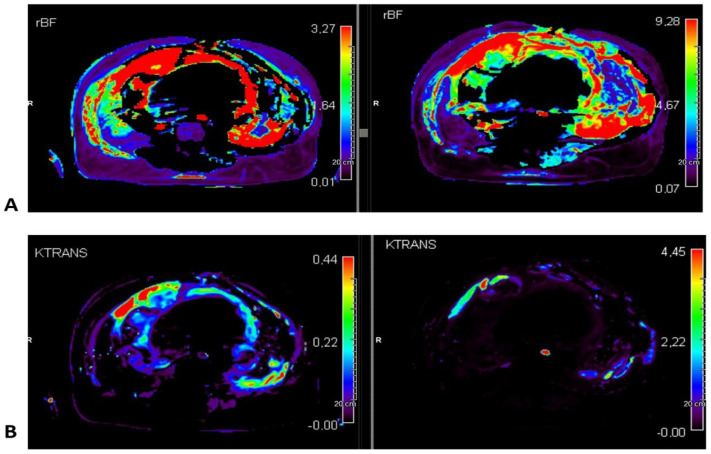
Perfusion (**A**) and permeability (**B**) maps in a patient with an inhomogeneous retroperitoneal liposarcoma that had disease progression. The post-treatment evaluation (on the right) shows increased dimension and higher perfusion values.

Generally, higher values of perfusion and permeability were detected in denser and more vascularized areas, with high discrepancies particularly in non-homogeneous lesions (Figure 1). Patients who had disease control showed overall higher pre-treatment permeability and lower perfusion and permeability after treatment. As an exception, the patient with WDLPS/DDLPS with a prevalent WDLPS component had no relevant radiological changes.

The same patient was not assessable regarding quantitative evaluation of perfusion and permeability because of technical issues pertaining to the radiology data. Moreover, more than half of the data about the precise biopsy site were missing (because an ultrasound-guided procedure had been performed, without available recorded imaging), so the graphic analysis of these data was not carried out.

**Figure 2:**
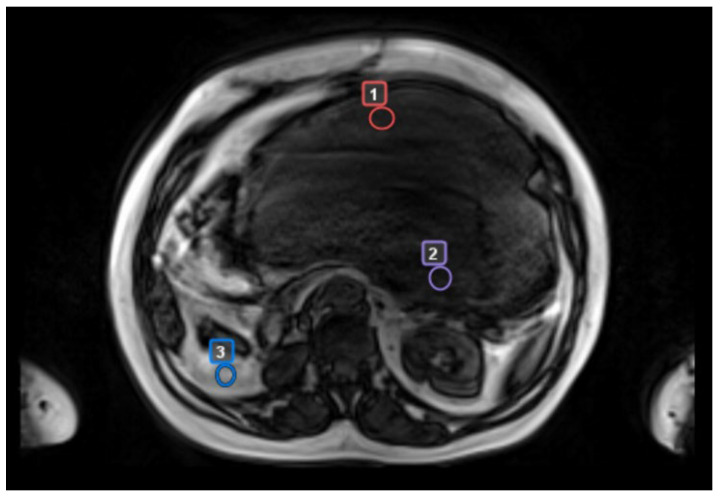
Example of ROI individuation in a patient with a voluminous retroperitoneal DDLPS. 1: “best” area; 2: “worst” area; 3: control area. ROI = region of interest; DDLPS = dedifferentiated liposarcoma.

We then analyzed separately the DCE-MRI perfusion and permeability values in assessable patients in “worst”, “best”, and control areas, respectively (ROIs, Figure 2).

**Figure 3:**
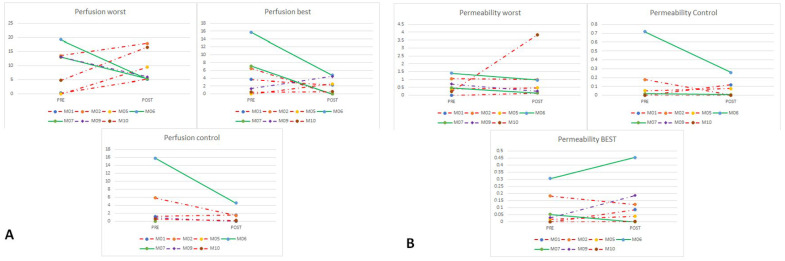
Perfusion (**A**) and permeability (**B**) evaluation in “worst” and “best” areas vs. control areas. Legend: patients with disease control are depicted in green; patients with progressive disease are depicted in red; the patient that had SD in the examined target lesions but had disease progression because of a new distant metastasis is depicted in purple.

Concerning perfusion in the “worst” areas, we found increased perfusion in patients with disease progression, while it decreased in patients with disease control. This change was not so clear in “best” areas, while perfusion values were generally lower in control areas and even lower after eribulin (Figure 3A). On the other hand, permeability decreased in patients with disease control both in “worst” and control areas, while in “best” areas a change was not evident (Figure 3B). Available perfusion and permeability data for each patient are detailed in the Supplementary Materials.

### 3.4. Pathology Assessment

Histological analysis was performed for the eight patients who had both pre- and post-treatment biopsies. We focused on the evaluation of the main morphological changes in cellularity, vascularization, stromal changes, and presence of cellular pleomorphism. Pathological features for each patient are summarized in Table 3.

**Figure 4:**
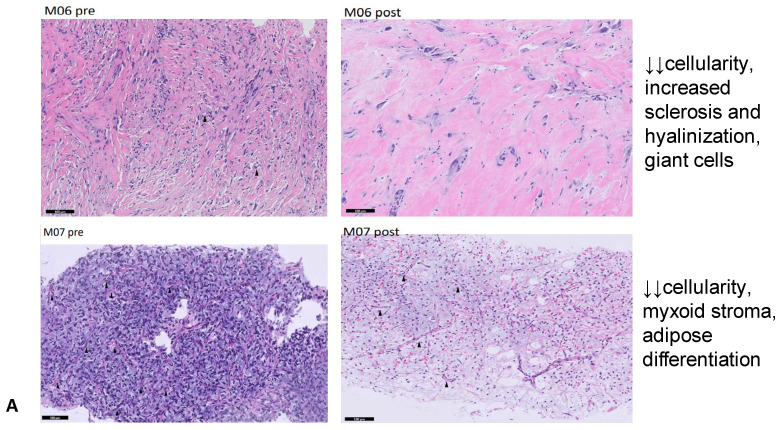

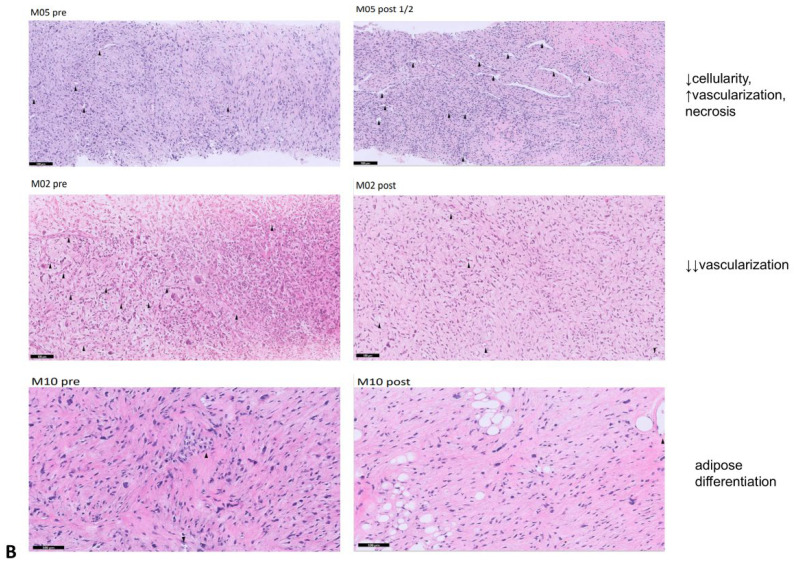
Post-treatment modifications (images on the **right**) compared to pre-treatment histology (on the **left**) in some responding (**A**) and non-responding (**B**) patients. Black arrows indicate blood vessels. Scale bar: 100 μm. ↑ = slight increase; ↓ = slight decrease; ↓↓ = significant decrease.

All patients demonstrated histological modifications except the patient with WDLPS/DDLPS with a prevalent WDLPS component. A decrease in cellular density was reported in 50% of patients (both in patients with RECIST disease control or PD), while a decrease in vascular structures was detected only in one patient. One patient had an increase in blood vessels and necrosis, in line with radiological PD. Interestingly, signs of tissue maturation with adipose differentiation were found in three (38%) patients (with lipoblasts in one patient), one of whom had RECIST disease progression. Significant examples of pathological changes are reported in Figure 4. Available pre- and post-treatment histological images are shown in the Supplementary Materials.

Clinical, radiological, and pathological outcomes are reviewed in Table 4. We considered disease control (PR or SD) vs. PD in radiological exams and signs of response vs. signs of worsening (for example increased vascularization) in pathology assessment. Overall, the mean concordance between CT scan (RECIST), DCE-MRI, and histology was 83.5%, with higher discrepancies between pathological and radiological data.

## 4. Discussion

In this study, we explored the mechanisms of action of eribulin in a cohort of patients with advanced pretreated LPS, combining clinical, radiological, and pathological assessments, with a particular emphasis on non-mitotic effects.

Our clinical results regarding eribulin effectiveness and toxicity were consistent with existing literature, except for a lower overall survival rate (mOS 8.7 months vs. 13.5 months in Schöffski et al. [12]). Patients in our study were highly pretreated and perhaps less fit than patients usually included in clinical trials. However, the patients that achieved disease control with eribulin (three patients, 27%) had numerically longer progression-free and overall survival (mOS 16.5 months), confirming that eribulin can be effective in some patients. This positive impact was also seen in patients with stable disease (SD), so any benefit was not exclusively related to radiologic tumor shrinking.

Focusing on the responding patient subset, we explored the underlying mechanisms of action integrating radiological and pathological response features. Considering changes in vascularization analyzed through DCE-MRI, we found reduced levels of tumor perfusion after eribulin treatment in patients that achieved disease control and elevated post-treatment tumor perfusion in patients with PD. Conversely, we did not find any patterns in permeability variations, perhaps because of the small sample size and tumor heterogeneity. Preclinical and clinical models of breast cancer previously showed that eribulin causes a reduction in global tumor hypoxia, increasing vascularization in hypoxic areas, and decreasing it in more vascularized areas, a unique rebalancing of tumor blood vessel structure and organization [19,20]. Our finding could be explained considering that DCE-MRI data are drawn from a single higher grade (more vascularized) ROI, so are coherent with preclinical results [19]. More comprehensive investigations analyzing the tumor mass as a whole will be useful to better quantify vascularization.

The observed effect on tumor perfusion may explain the positive impact of eribulin on OS without affecting PFS [12,16]. It is well recognized that tumor vessels present altered structure and modified distribution compared with normal tissue [21], being vascularization is a crucial factor for tumor growth [22]. These alterations appear to favor the establishment of a tumor microenvironment (TME) that selects more aggressive tumor cells, attracts immunosuppressive cells, and compromises drug delivery [23].

While other anti-microtubule drugs cause disruption of the tumor vascular network and inhibit neoangiogenesis [24,25], eribulin normalizes tumor blood vessels’ structure and organization [19,20]. In order to support a hypothetical role of anti-antiangiogenetic drugs in STS, pazopanib provides a model. Pazopanib is the regulator-approved kinase inhibitor for soft tissue sarcomas, but LPS patients were excluded from the pivotal phase III PALETTE trial [26] based on a prior negative phase II trial [27]. However, following a centralized pathological review, two patients were reclassified and assigned to the liposarcoma cohort [27,28]. On the basis of this recategorization, pazopanib achieved the clinical activity cutoff for liposarcoma in the phase II trial [27,28], consistent with other phase II studies in which activity was seen in liposarcoma patients [29,30,31]. Taking into account these data, vascularization appears to be a targetable feature even in LPS. Our exploratory findings on correlation between tumor perfusion modifications induced by eribulin and tumor response support this hypothesis and will benefit from a larger study to confirm or refute this finding.

Epithelial-to-mesenchymal transition (EMT), the acquisition of mesenchymal characteristics in epithelial tumor cells, also plays an important role in metastatic potential [32,33]. In preclinical models and in a clinical study in breast cancer, eribulin induced an inversion of EMT reducing tumor migration and lung metastasis [34]. In sarcoma models, preclinical data showed an upregulation of adipocytic differentiation genes in liposarcoma cells after exposure to eribulin [20]. Our pathological assessment showed histological changes in all patients except for the one with WDLPS/DDLPS with a prevalent WDLPS component; we detected post-treatment morphological changes as the presence of lipoblasts and mature fat tissue features (lower grade). After treatment, we also observed a decrease in cellularity, and blood vessels changes in some patients, which did not always align with the radiological response. This discordance could be explained by tumor heterogeneity, but it can be hypothesized that modifications in both vascularization and tumor cells phenotype are necessary to achieve tumor control.

The observed pathological variations in tumor tissue and microenvironment could improve sensitivity to other therapies, suggesting a role for eribulin in earlier lines of treatment or in novel combinations. Preclinical studies showed favorable synergistic effects in association with several chemotherapeutic or targeted agents [18,35]. Based on our preliminary results, earlier administration of eribulin and/or a specific sequential treatment plan provides novel future treatment strategies to pursue.

Our study has several limitations. The small sample size and long accrual time limited us to descriptive analyses with only modest differences in study procedures such as DCE-MRI. A larger sample size with longer follow-up and more sensitive testing, such as single cell transcription analysis, would be a natural extension to this hypothesis-generating study.

## 5. Conclusions

Our exploratory study further supports the thesis of non-mitotic effects of eribulin on vascularization and cellular differentiation in LPS patients. As a future perspective unveiled by our study, a transcriptomic analysis in bulk tumor or in single cells could not just confirm the impact of eribulin on fat tissue differentiation and vascularity but also uncover new non-mitotic effects of eribulin. DCE-MRI appears to be a useful tool to better understand functional characteristics of tumors, especially in large heterogeneous masses as seen in sarcoma patients. Further studies are needed to confirm these results and identify patients who will benefit the most from eribulin therapy.

## Figures and Tables

**Table 1:** Patient characteristics. FNCLCC = Fédération Nationale des Centres de Lutte le Cancer; DDLPS = dedifferentiated liposarcoma; WDLPS = well-differentiated liposarcoma; MLPS = myxoid liposarcoma; EI = epirubicin + ifosfamide; trabe = trabectedin; AI = doxorubicin + ifosfamide; A = doxorubicin; ifo = ifosfamide; gem = gemcitabine. * Tumor grade was not specified in the original pathology report; however, the finding of dedifferentiated liposarcoma in those two specimens means the tumor was either grade 2 or 3; these are both considered high grade lesions.

Patient ID	Age at Diagnosis	Gender	Histology	Primary	Stage at Diagnosis	Grade (FNCLCC Grading System) at Diagnosis	Disease Sites at Enrollment	Prior Treatments	Time (Months) from Diagnosis to Eribulin Treatment
M01	60	F	DDLPS (spindle cells in myxoid stroma)	retroperitoneum	III	G2 or G3 *	abdomen, soft tissues	EI × 2/trabe × 9	22
M02	44	F	DDLPS (lipoblastic differentiation)	retroperitoneum	III	G3	abdomen	AI × 3/trabe × 10	94
M03	59	M	WDLPS/ DDLPS (focal hypercellularity)	retroperitoneum	III	G2 or G3 *	abdomen	trabe × 4	91
M04	71	M	DDLPS (spindle and pleomorphic cells in myxoid stroma)	retroperitoneum	III	G3	abdomen, bone, lung	A × 5/trabe × 4	90
M05	34	F	DDLPS (spindle and pleomorphic cells)	extremities	III	G2	lung, soft tissues	AI × 6/ifo × 3/trabe × 7	40
M06	42	F	DDLPS (spindle and pleomorphic cells)	retroperitoneum	III	G3	abdomen	trabe × 4/ifo × 2	64
M07	28	F	MLPS (high grade)	extremities	III	G3	abdomen, bone	AI × 5/trabe × 22/ ifo × 3/gem × 3	128
M08	80	M	DDLPS (spindle and pleomorphic cells in myxoid stroma)	mediastinum	III	G3	lung, liver	ifo × 6/trabe × 4	18
M09	67	M	MLPS (high grade)	extremities	III	G3	bone, soft tissues	trabe × 40/ifo × 3	70
M10	42	M	DDLPS (myxofibrosarcoma-like component)	retroperitoneum	III	G3	abdomen	trabe × 8/ifo × 6	123
M11	63	M	DDLPS (spindle and pleomorphic cells)	retroperitoneum	III	G3	lung	A × 3/ifo × 5	49

**Table 2:** DCE-MRI assessment. DDLPS = dedifferentiated liposarcoma; WDLPS = well-differentiated liposarcoma; MLPS = myxoid liposarcoma; PD = progressive disease; SD = stable disease; PR = partial response. Legend: patients with patient ID in red had PD, the ones in green had SD or PR, the one in blue had SD in the examined target lesions but had disease progression because of a new distant metastasis. The worst nodules were defined as tumor areas with radiologic features suggestive of the most aggressive biology (reduction in apparent diffusion coefficient (ADC) maps, high contrast enhancement, low T2 signal).

Patient ID	Histology	Target Lesions Site	Pre-Treatment General Assessment	Pre-Treatment Perfusion	Pre-Treatment Permeability	Post-Treatment General Assessment	Post-Treatment Perfusion	Post-Treatment Permeability
M01	DDLPS	abdomen	one worst nodule in bulky mass	high in worst nodule	high in worst nodule	PD (worst nodule no more detectable, overall increased bulky mass)	significantly higher	stable
M02	DDLPS	abdomen	homogeneous mass	high in 2 regions	high in 2 regions	PD (1 nodule increased, 1 nodule stable)	higher	significantly lower in stable nodule, remains high in increased nodule
M03	WDLPS/ DDLPS	abdomen	homogeneous multifocal masses	low	low	SD	stable (low)	stable (low)
M05	DDLPS	thigh	all high-grade nodule	high in rim, low in center (necrosis)	NA	PD (overall dimension)	stable (high in rim, low in center—necrosis)	high (no comparison available)
M06	DDLPS	abdomen	one worst nodule, multifocal disease	very high	very high	PR	significantly lower	significantly lower
M07	MLPS	abdomen	homogeneous nodules	low	low	SD	lower	lower
M09	MLPS	trunk	homogeneous nodules	high in rim, low in center (necrosis)	low	SD	lower	lower
M10	DDLPS	abdomen	two worst nodules in bulky mass	high in 2 worst nodules, low in center (necrosis)	high in 2 worst nodules rims	PD (increased worst nodules dimension)	high in 2 worst nodules rims, low in the rest of the mass	stable (high in 2 worst nodules rims, low in the rest of the mass)

**Table 3:** Histologic assessment. DDLPS = dedifferentiated liposarcoma; WDLPS = well-differentiated liposarcoma; MLPS = myxoid liposarcoma. Legend: patients with patient ID in red had PD, and the ones in green had SD or PR, the one in blue had SD in the examined target lesions but had disease progression because of a new distant metastasis.

Patient ID	Histology	Pre-Treatment Assessment	Post-Treatment Assessment
M01	DDLPS	Myxoid stroma, low cellularity, high vascularization	Significant cellularity reduction
M02	DDLPS	Many blood vessels, uneven cellularity, pleomorphic cells	Significantly less vascularized
M03	WDLPS/DDLPS	Adipose tissue with slightly higher cellularity (WDLPS)	No significant variations
M05	DDLPS	High cellularity	Lower cellularity, more blood vessels, necrosis
M06	DDLPS	High cellularity, scarce pleomorphism	Significant cellularity reduction, increased sclerosis and hyalinization, giant cells
M07	MLPS	High cellularity, many blood vessels	Significant cellularity reduction, more myxoid stroma, lipoblasts and signs of adipose differentiation
M09	MLPS	Homogeneous MLPS	Increased sclerosis and hyalinization and signs of adipose differentiation
M10	DDLPS	High necrosis, no lipoblasts	No necrosis, signs of adipose differentiation

**Table 4:** Overall response assessment. DDLPS = dedifferentiated liposarcoma; WDLPS = well-differentiated liposarcoma; MLPS = myxoid liposarcoma; PD = progressive disease; SD = stable disease; PR = partial response; NA = not assessable. Legend: patients with patient ID in red had PD, the ones in green had SD or PR, the one in blue had SD in the examined target lesions but had disease progression because of a new distant metastasis. ↑ = slight increase; ↓ = slight decrease; ↓↓ = significant decrease.

Patient ID	Histology	Protocol Completion	Total Eribulin Cycles	RECIST Response (Target Lesions)	DCE-MRI Response	Histology Changes	Concordance Between RECIST, MRI and Histology
M01	DDLPS	yes	4	PD (29 × 24 cm vs. 19 × 15 cm)	PD	↓↓ cellularity	67%
M02	DDLPS	yes	4	PD (7 × 6.7 cm vs. 5.8 × 3.5 cm, 6 × 5 cm vs. 3.8 × 3.5 cm)	PD	↓↓ vascularization	67%
M03	WDLPS/ DDLPS	yes	3 years	SD (6.5 × 4.8 cm vs. 7.5 × 4.8 cm)	SD	none	100%
M04	DDLPS	no	4	PD (10 × 8 cm vs. 8.3 × 3.5 cm)	NA	NA	NA
M05	DDLPS	yes	4	PD (9.6 × 5.4 cm vs. 6.5 × 4.3 cm)	PD	↓ cellularity, ↑ vascularization, necrosis	100%
M06	DDLPS	yes	9	SD (2.8 × 2 cm vs. 2.5 × 1.8 cm, 4.5 × 4.3 cm vs. 4.9 × 4.8 cm)	PR	↓↓ cellularity, increased sclerosis and hyalinization, giant cells	100%
M07	MLPS	yes	8	PR (8.7 × 5 cm vs. 13.9 × 64, 6.3 × 4.4 cm vs. 7.8 × 7.5 cm)	SD	↓↓ cellularity, myxoid stroma, adipose differentiation	100%
M08	DDLPS	no	2	PD (new multiple lung and mediastinum lesions, increased dimension of prior lesions)	NA	NA	NA
M09	MLPS	yes	4	PD (new abdominal lesion, SD in target lesions: 9.5 × 8.2 cm vs. 9.8 × 7.8 cm, 5.8 × 3.9 cm vs. 5.6 × 4.2 cm)	SD	Increased sclerosis and hyalinization, adipose differentiation	67%
M10	DDLPS	yes	3	PD (9.8 × 5.3 cm vs. 6.9 × 4.9 cm, 7.9 × 4.6 cm vs. 6.5 × 4.9 cm)	PD	adipose differentiation	67%
M11	DDLPS	no	1	PD (new clinically significant pleural effusion)	NA	NA	NA

## Data Availability

The raw data supporting the conclusions of this article will be made available by the authors on request, according to the data protection laws.

## Supplementary Materials

The following supporting information can be downloaded at: https://www.mdpi.com/article/10.3390/cancers17060976/s1.
