# Supplementary material for: Assessment of the Mechanisms of Action of Eribulin in Patients with Advanced Liposarcoma Through the Evaluation of Radiological, Functional, and Tissue Responses: A Prospective Monocentric Study (Malibu Study)
Source: Cancers (Basel). 2025 Mar 13;17(6):976. doi: 10.3390/cancers17060976 (PMC11940360; doi:10.3390/cancers17060976)
Supplement: Supplementary file 1 [file cancers-17-00976-s001.zip › Supplementary Material Malibu study.pdf]

SUPPLEMENTARY MATERIAL

Here we present pre- and post-treatment histological images and diffusion contrast-enhanced magnetic resonance imaging (DCE-MRI) perfusion and permeability quantitative evaluations (when available) for each patient.

M01

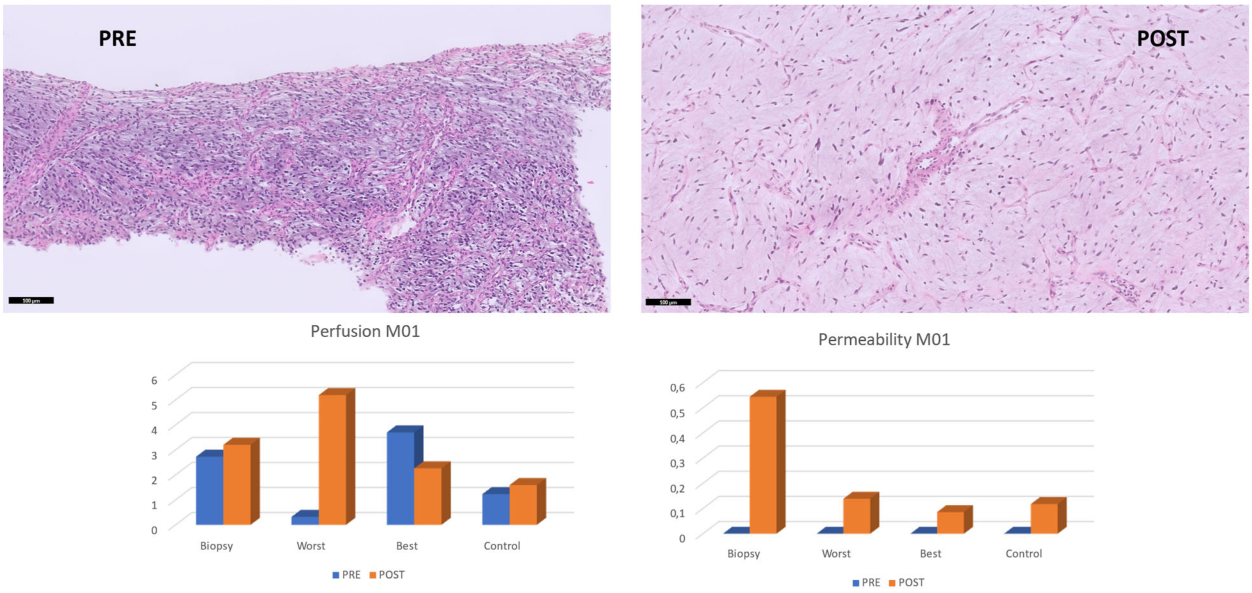

M02

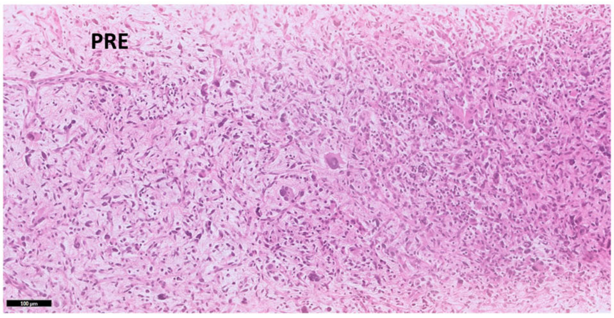

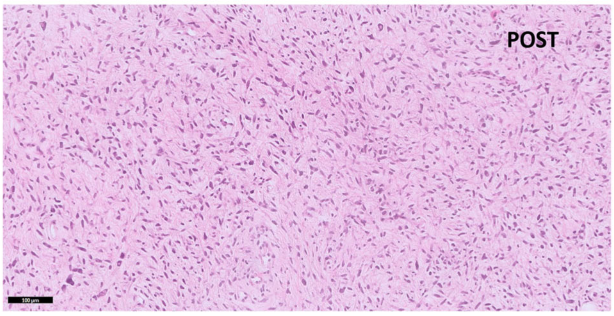

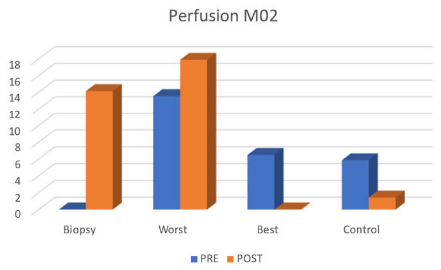

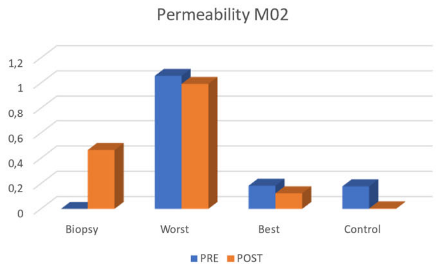

M03

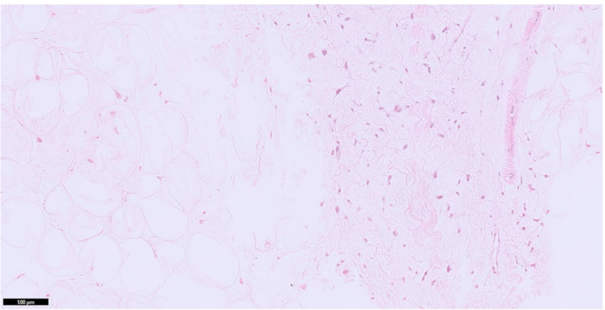

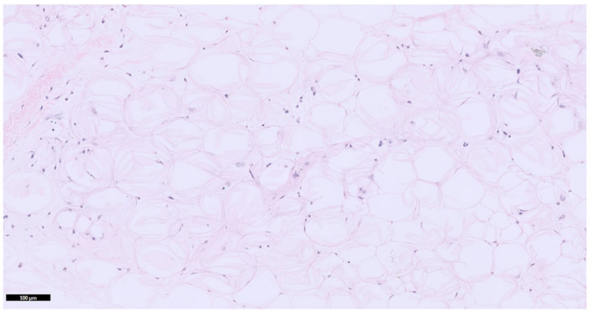

Perfusion and permeability NA

M05

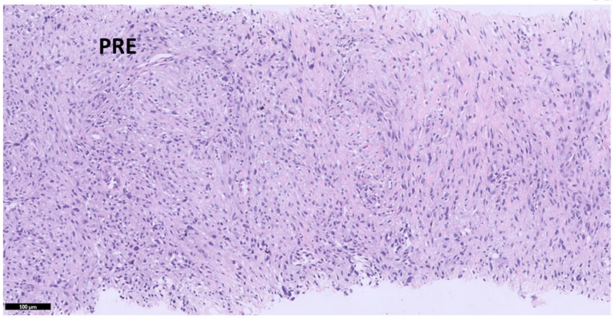
PRE

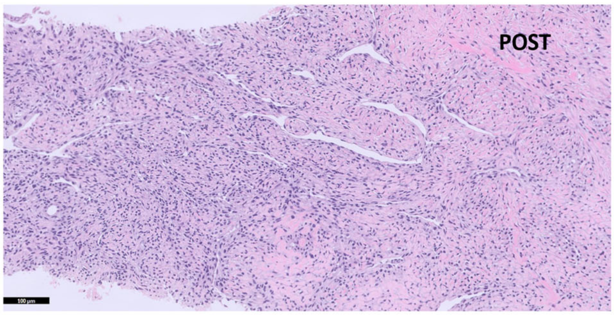
POST

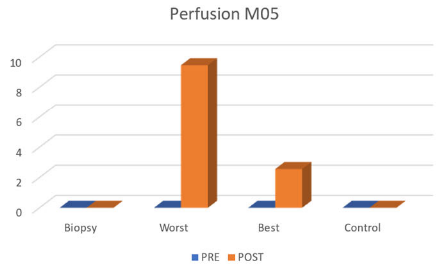
Perfusion M05
10
8
6
4
2
0
Biopsy
Worst
Best
Control
PRE
POST

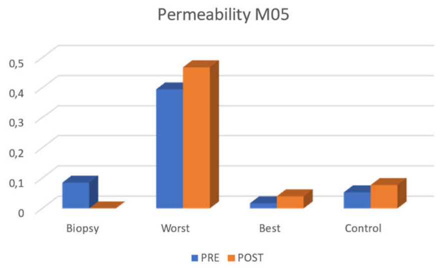
Permeability M05
0,5
0,4
0,3
0,2
0,1
0
Biopsy
Worst
Best
Control
PRE
POST

M06

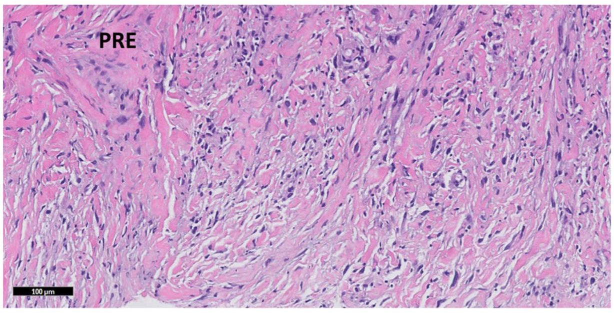
PRE

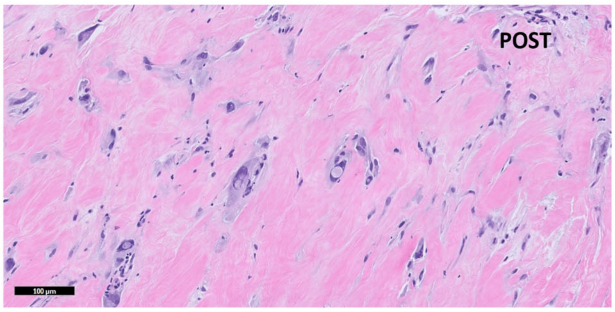
POST

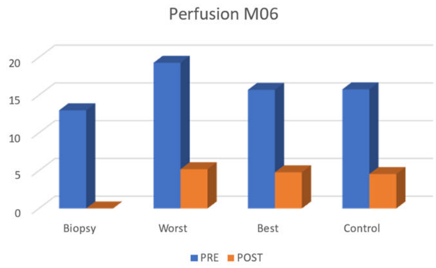
Perfusion M06
20
15
10
5
0
Biopsy
Worst
Best
Control
PRE
POST

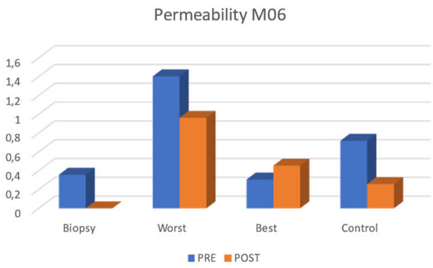
Permeability M06
1,6
1,4
1,2
1
0,8
0,6
0,4
0,2
0
Biopsy
Worst
Best
Control
PRE
POST

M07

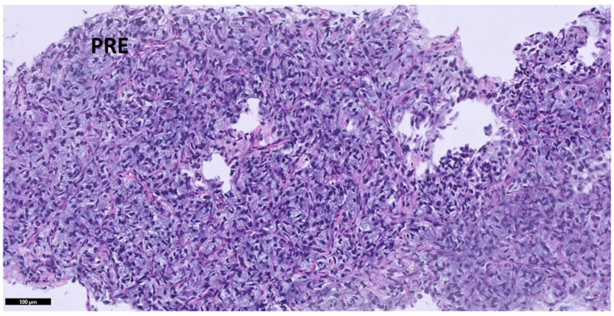
PRE

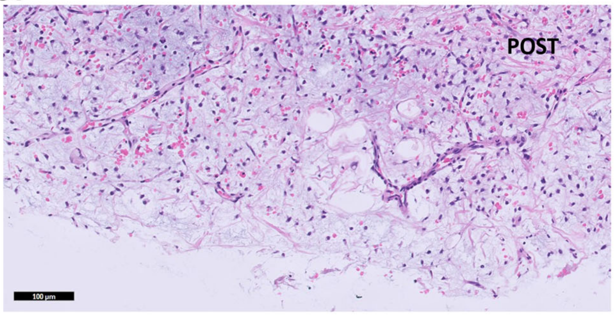
POST

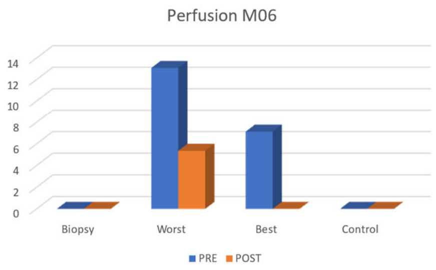
Perfusion M06
14
12
10
8
6
4
2
0
Biopsy
Worst
Best
Control
PRE
POST

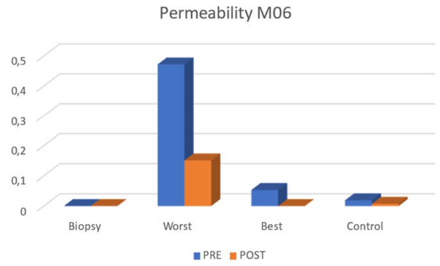
Permeability M06
0,5
0,4
0,3
0,2
0,1
0
Biopsy
Worst
Best
Control
PRE
POST

M09

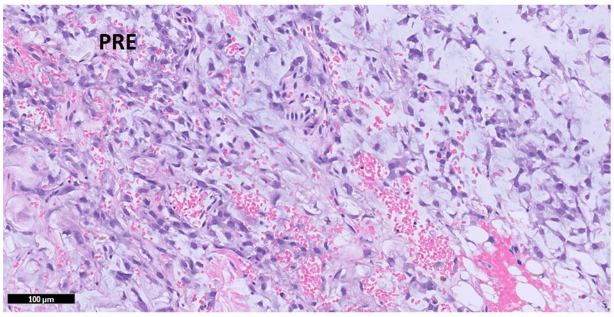
PRE
100 µm

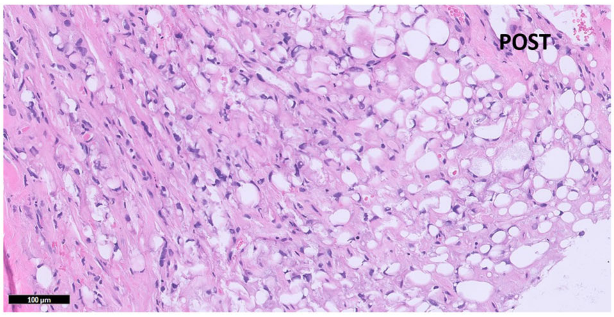
POST

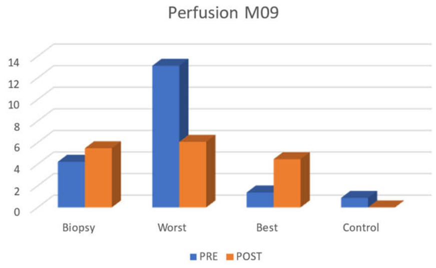
Perfusion M09
14
12
10
8
6
4
2
0
Biopsy
Worst
Best
Control
PRE
POST

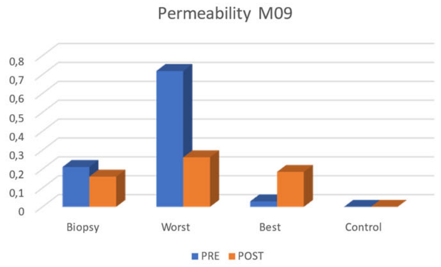
Permeability M09
0,8
0,7
0,6
0,5
0,4
0,3
0,2
0,1
0
Biopsy
Worst
Best
Control
PRE
POST

M10

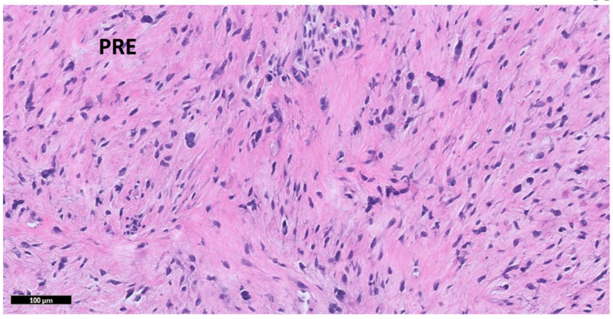
PRE
100 µm

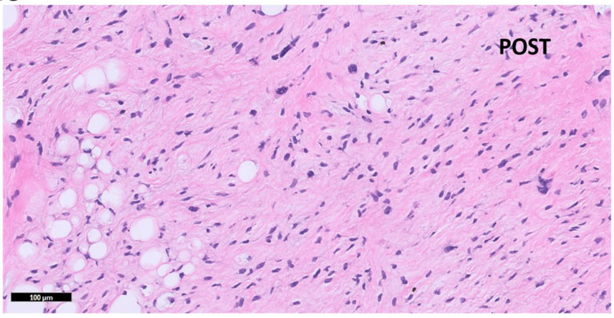
POST
100 µm

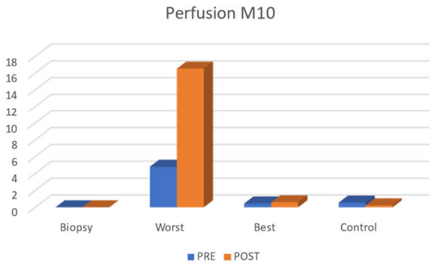
Perfusion M10
18
16
14
12
10
8
6
4
2
0
Biopsy
Worst
Best
Control
PRE
POST

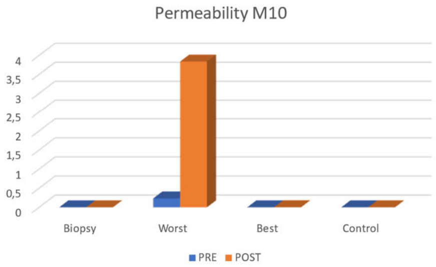
Permeability M10
4
3,5
3
2,5
2
1,5
1
0,5
0
Biopsy
Worst
Best
Control
PRE
POST
