# Supplementary figures and images for: Assessment of the Mechanisms of Action of Eribulin in Patients with Advanced Liposarcoma Through the Evaluation of Radiological, Functional, and Tissue Responses: A Prospective Monocentric Study (Malibu Study)
Source: Cancers (Basel). 2025 Mar 13;17(6):976. doi: 10.3390/cancers17060976 (PMC11940360; doi:10.3390/cancers17060976)

M02 pre

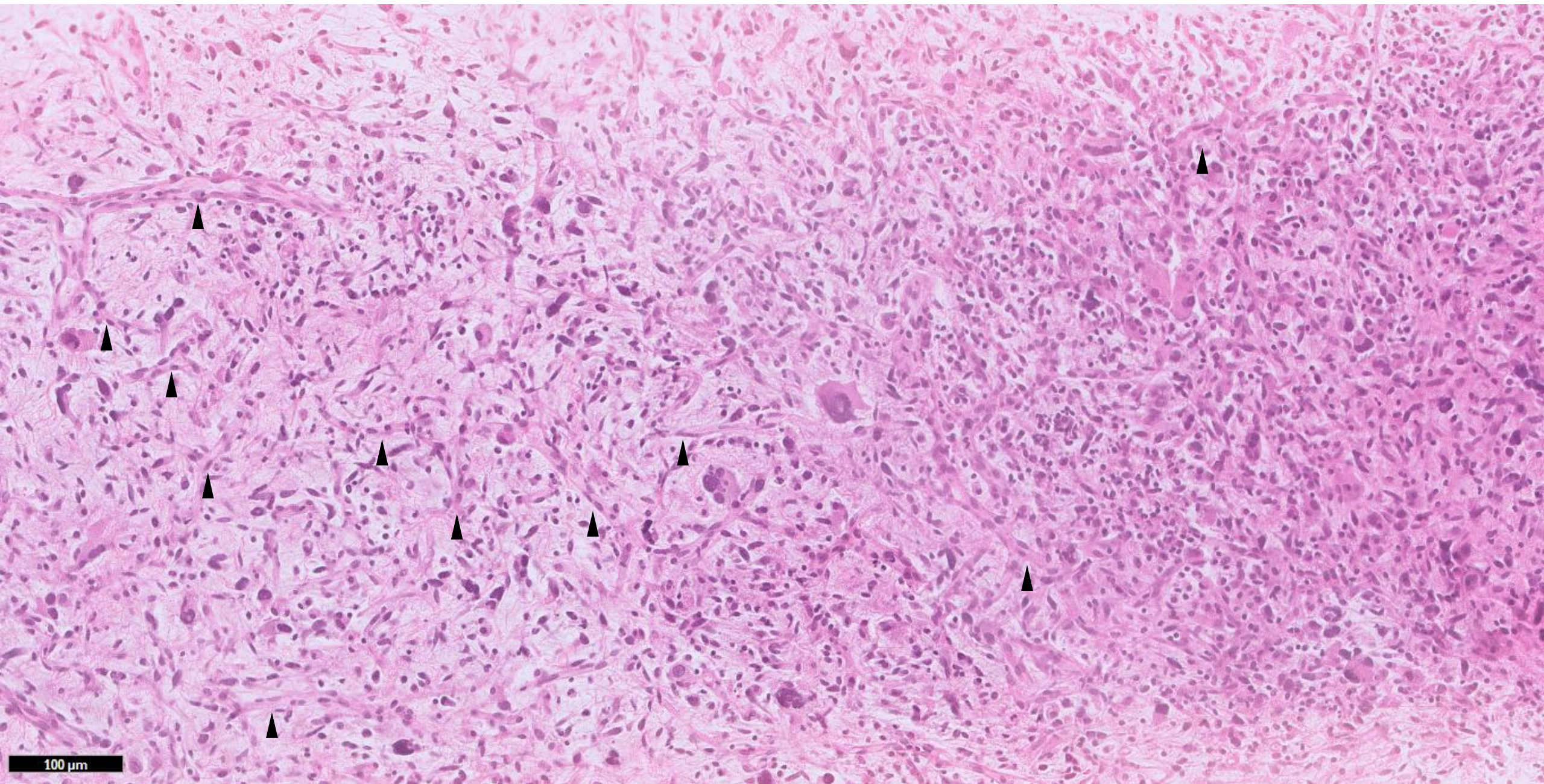

M02 post

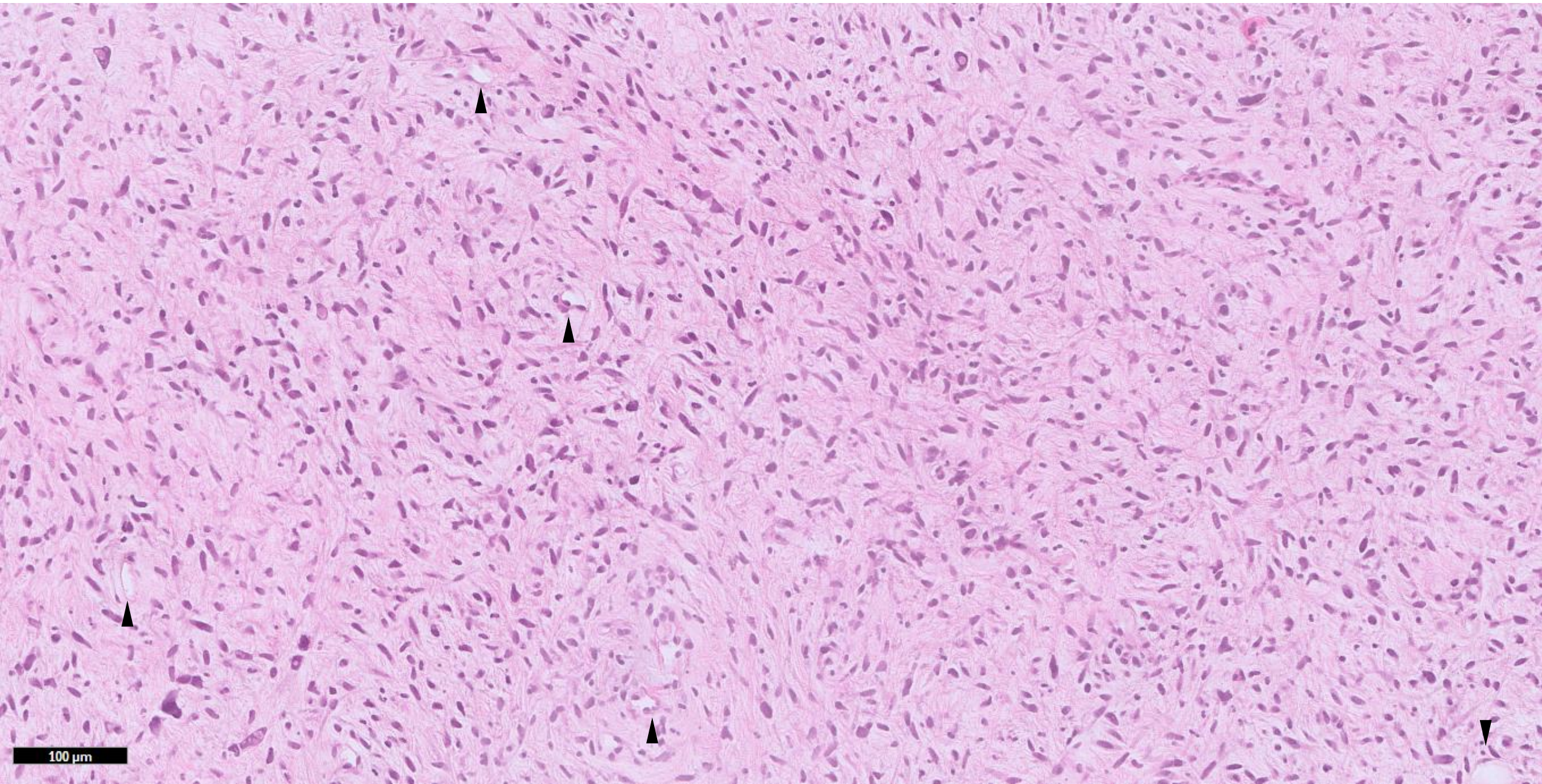

M05 pre

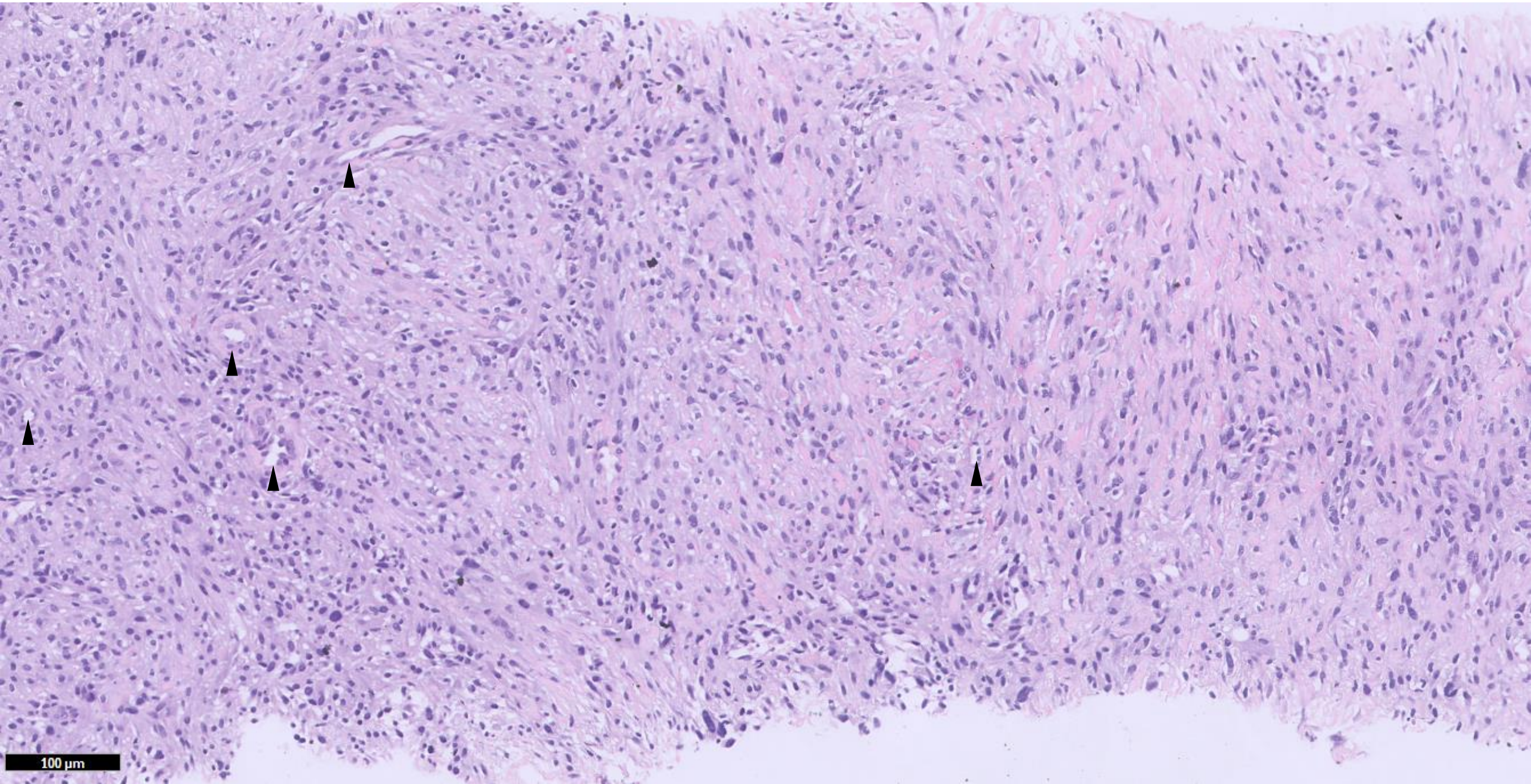

M05 post 1/2

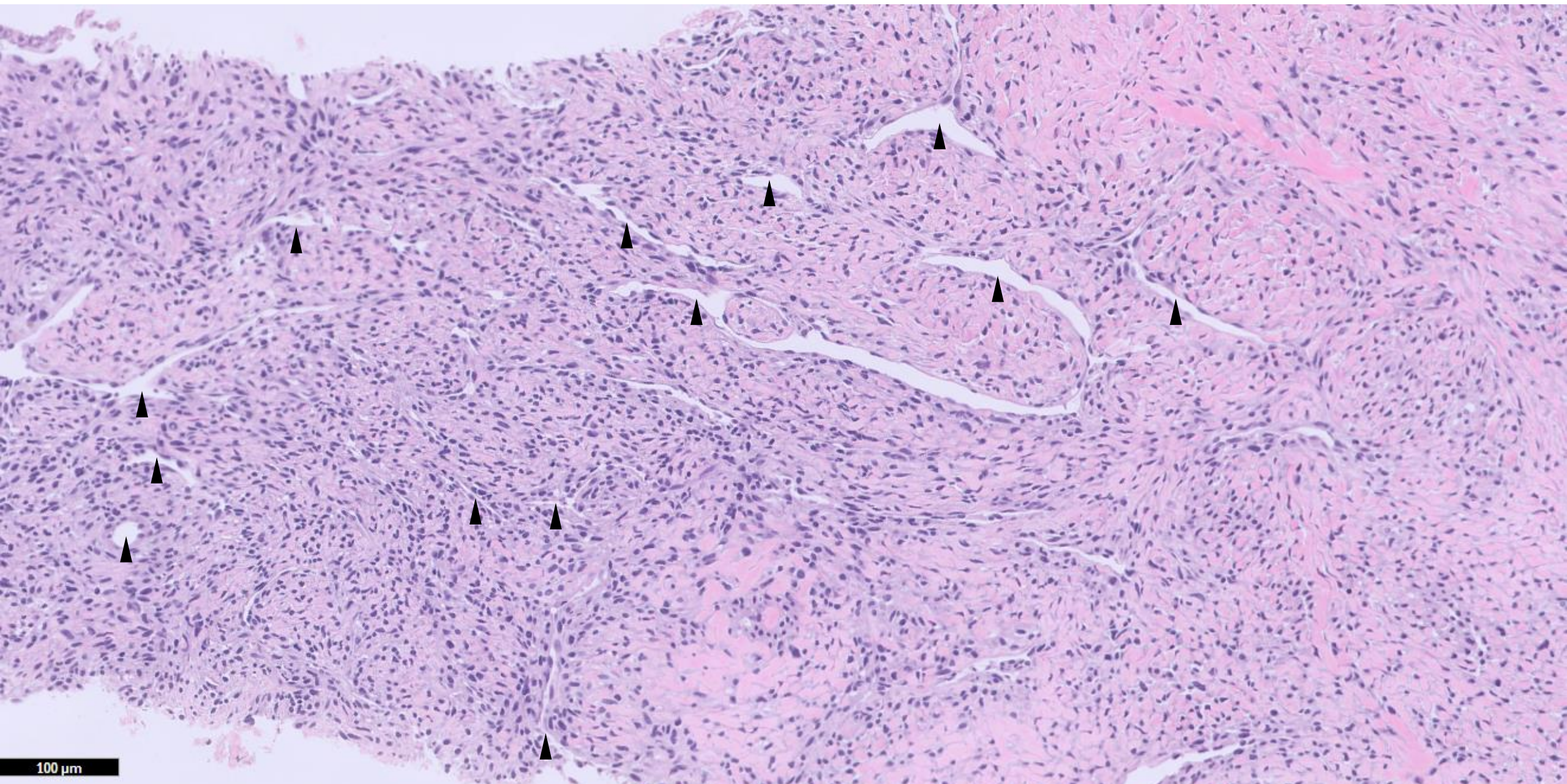

100  $\mu$ m

# M05 post ½ necrosis

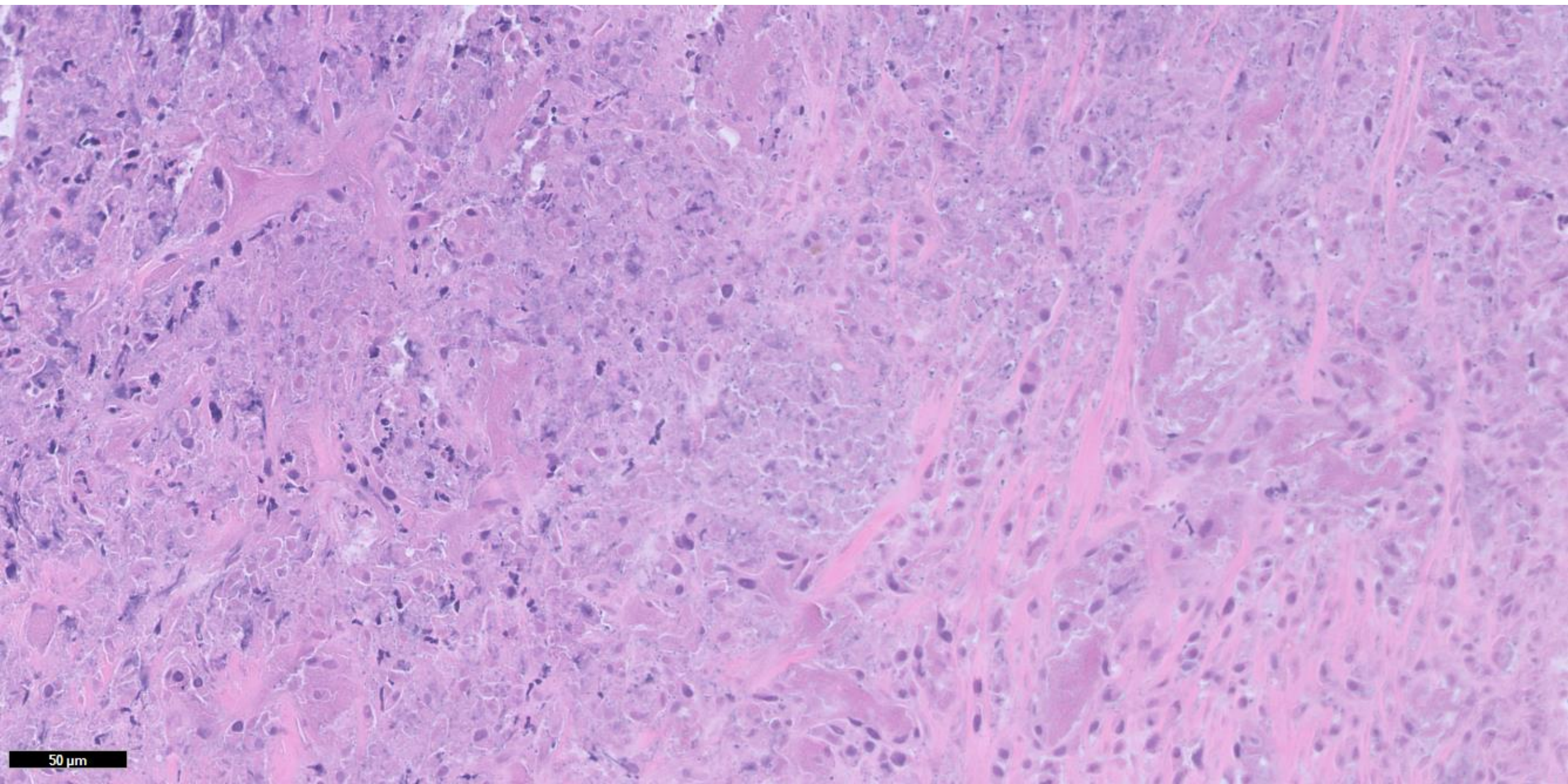

M06 pre

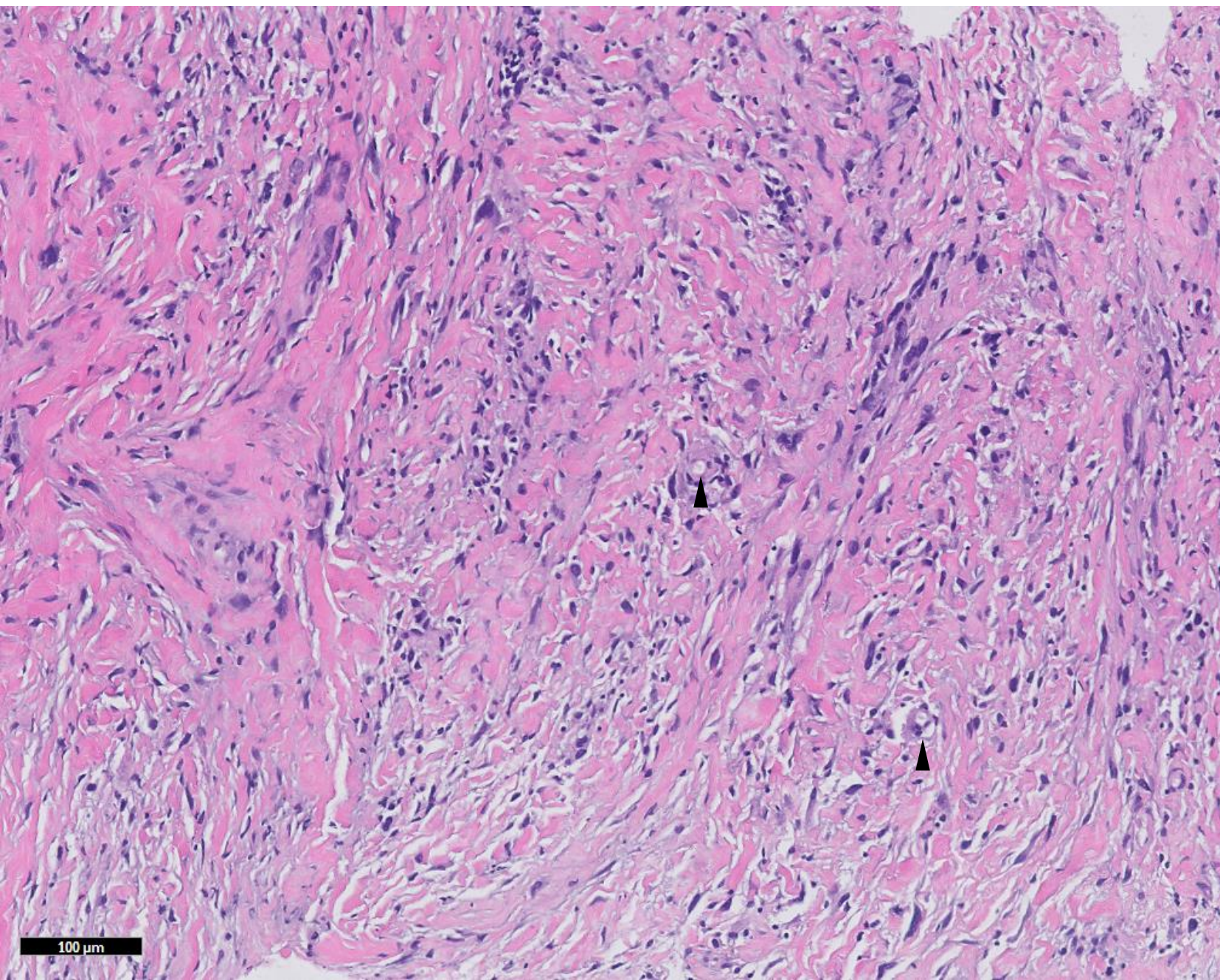

M06 post

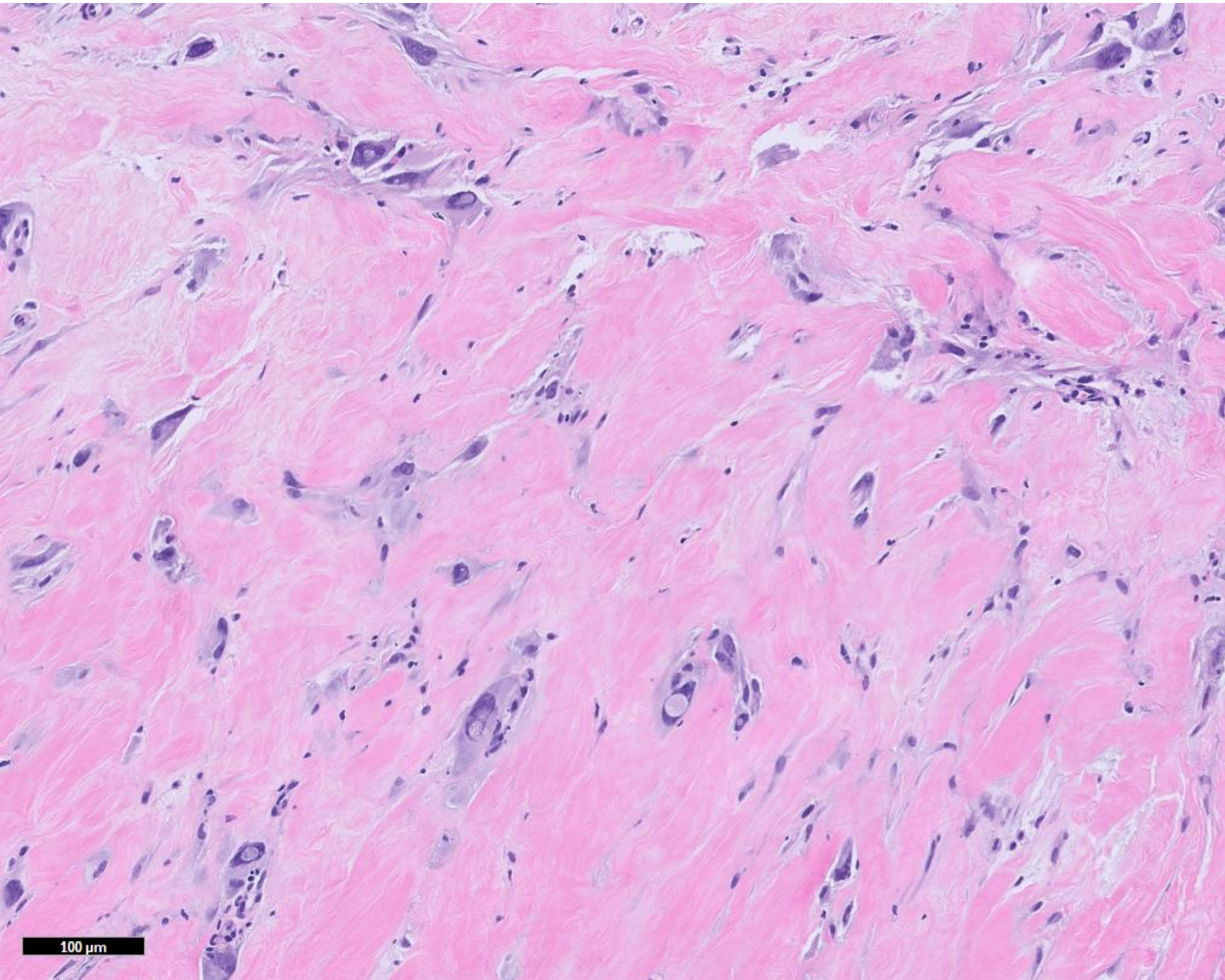

M07 pre

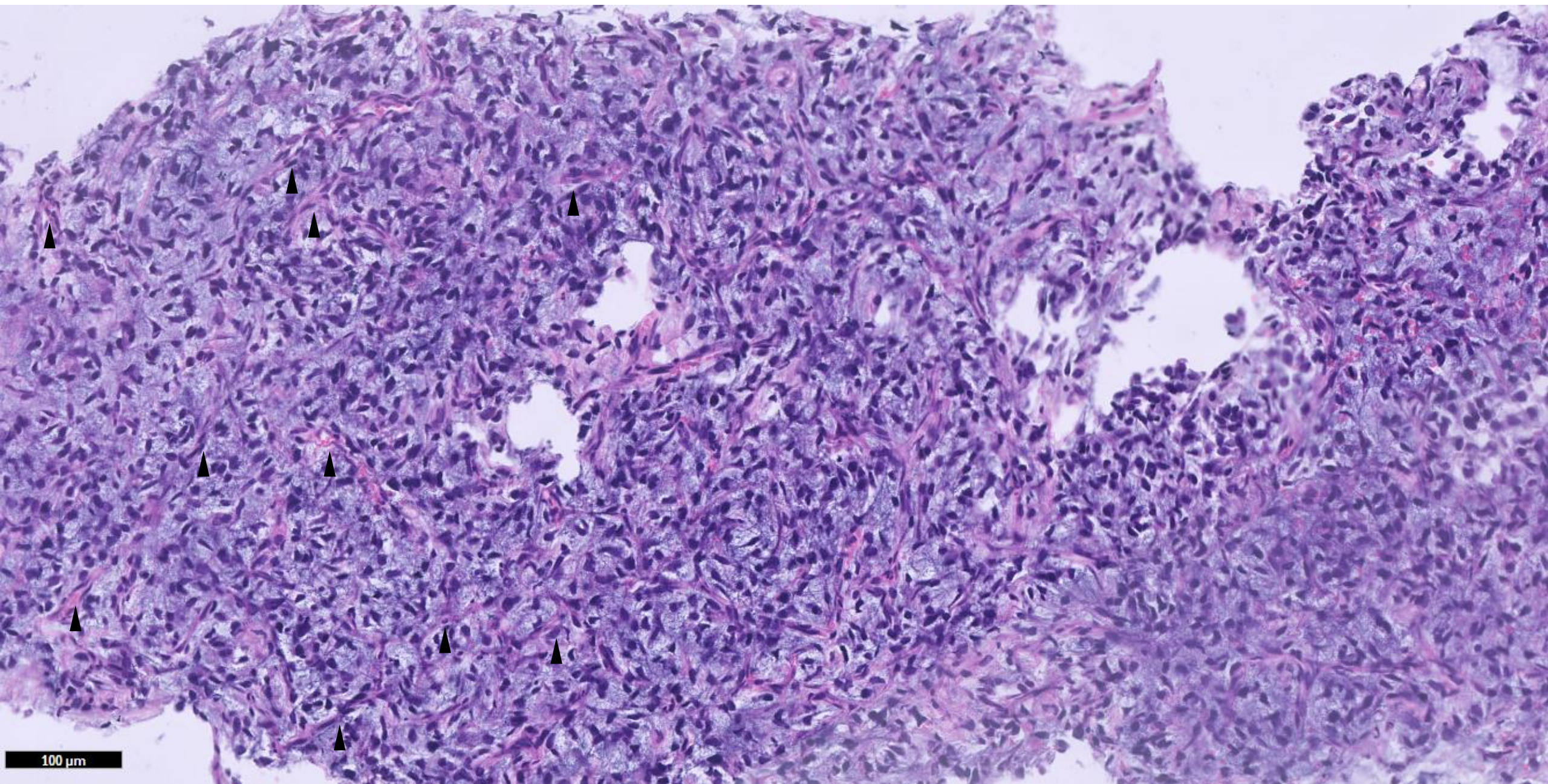

M07 post

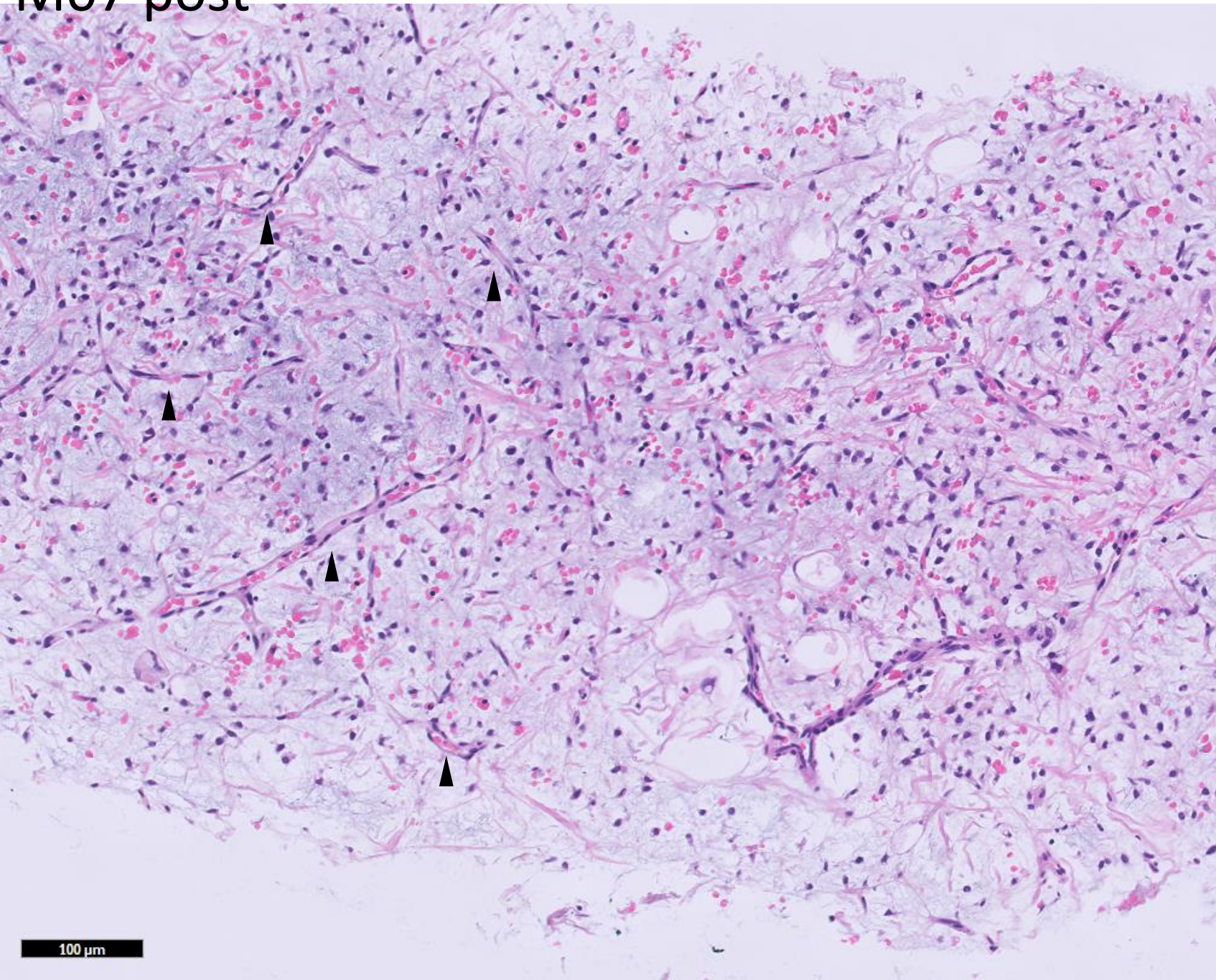

M10 pre

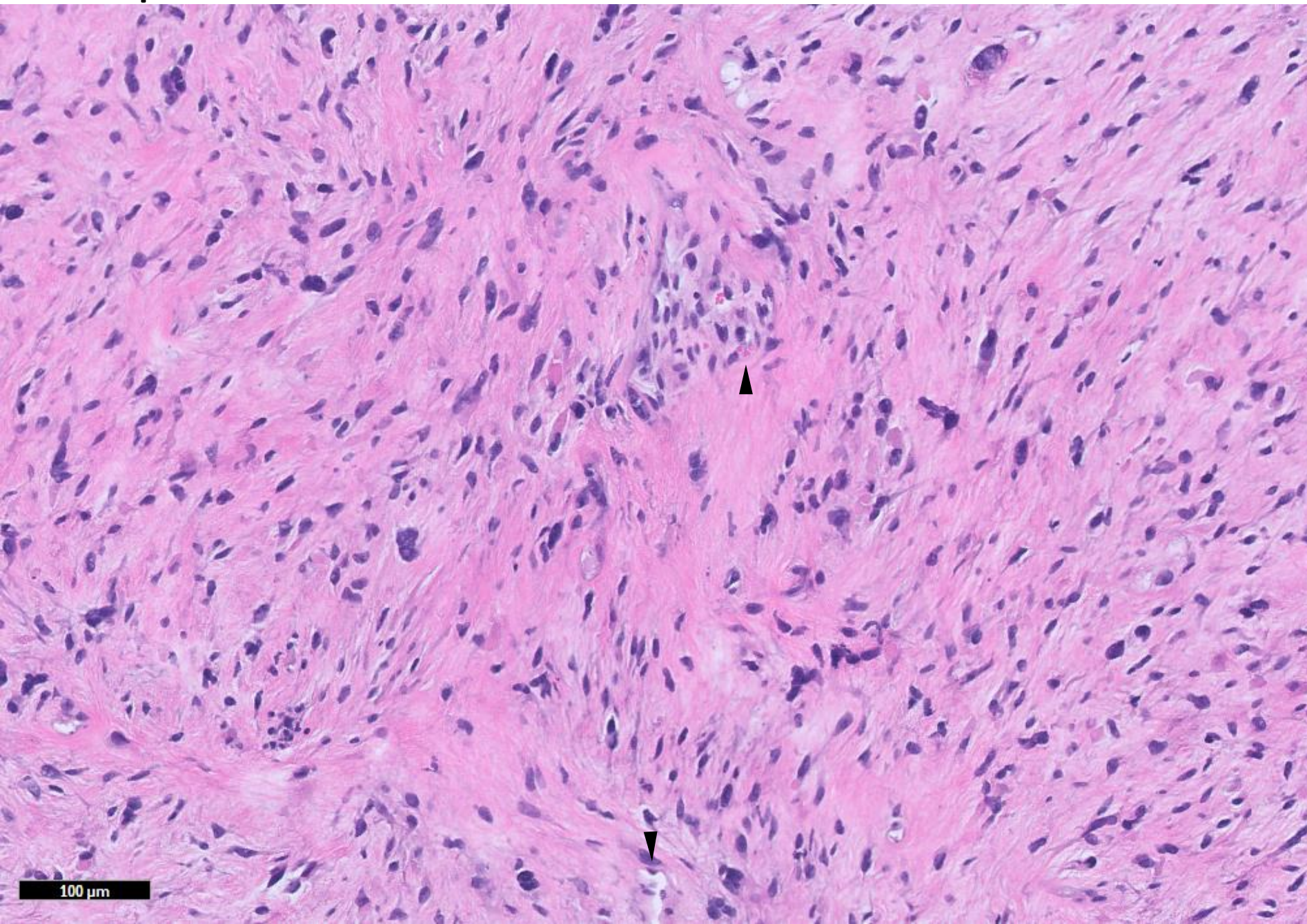

M10 post

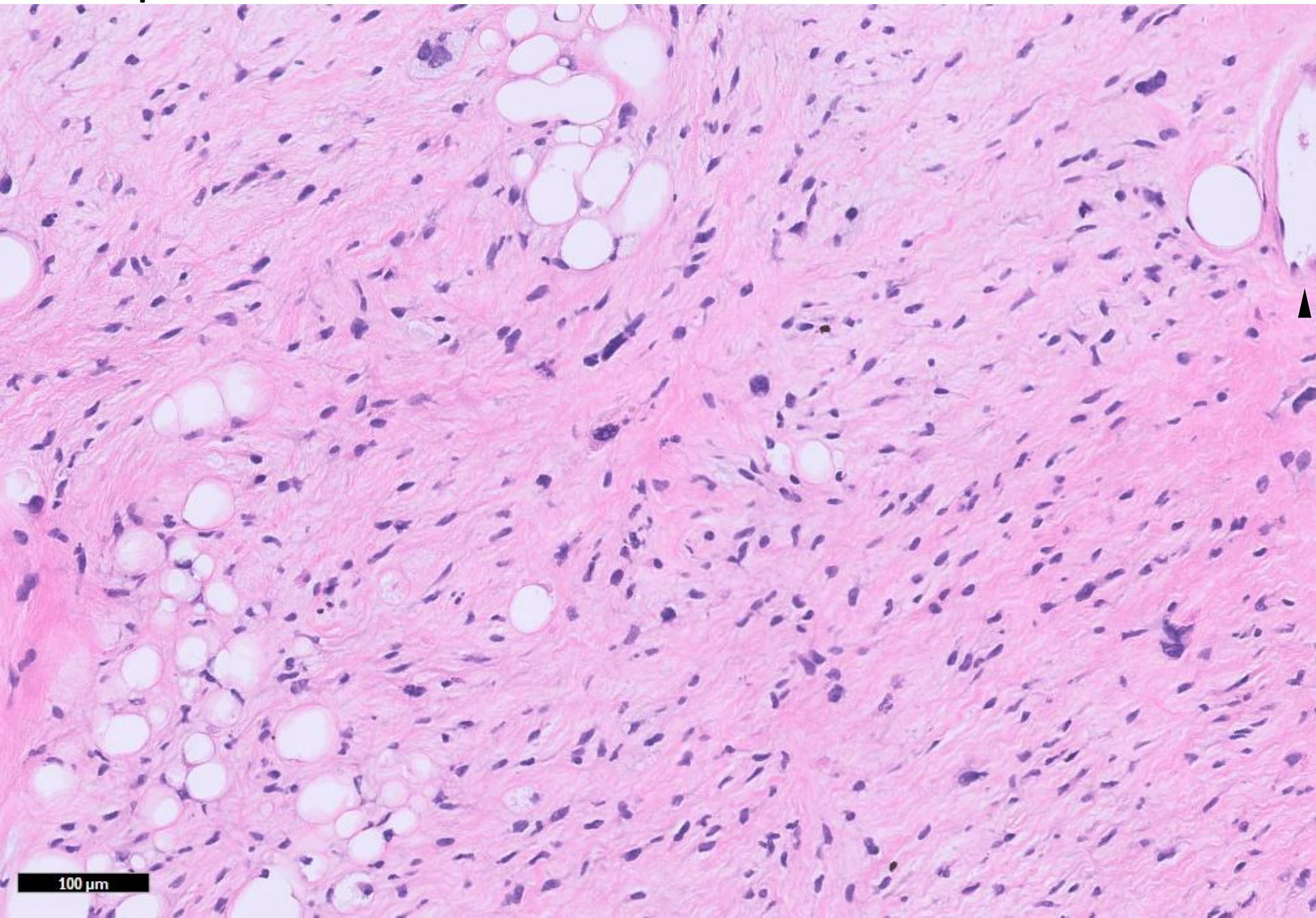

Supplement: Supplementary file 1 [file cancers-17-00976-s001.zip › Histology high quality Supplementary images.pdf]
